# The perils of penile enhancement: case report of a fulminant penile infection

**DOI:** 10.1186/s12894-021-00878-5

**Published:** 2021-08-24

**Authors:** Nicole Wen Mun Khor, Ankur Dhar, Alistair Cameron-Strange

**Affiliations:** 1 Murnaghan Urology Department, Prince of Wales Hospital, Sydney, Australia; 2 Prince of Wales Clinical School, UNSW Medicine, Sydney, NSW Australia

**Keywords:** Penile diseases, Urologic diseases, Penis, Infections, Hyaluronic acid, Injections, Case report

## Abstract

**Background:**

Penile enhancement with injectable agents is a rising trend and yet has received little scientific attention despite the potential for serious complications. These include cosmetic, functional and systemic complications that may require complex penile reconstructive surgery. We report a case of delayed severe infection following penile filler insertion leading to multi-organ failure and intensive care support.

**Case presentation:**

A 31-year-old man presented with fevers and progressive pain and swelling of the penile shaft, 3 days after unprotected sexual intercourse. The patient received subcutaneous hyaluronic filler injections at a cosmetic clinic for penile enlargement two months prior to presentation. Relevant social history include polysubstance abuse and multiple sexual partners. Physical examination revealed gross penile oedema and erythema, with a ventral curvature of the penile shaft and a superficial abrasion on the distal ventral penile shaft. Within 24 h the patient developed septic shock with anuria, hypotension and fevers to 40 °C, requiring transfer to the Intensive Care Unit (ICU) for vasopressor and inotropic support. Intraoperative penile exploration revealed multiple pus stained fillers which were drained and grew Streptococcus Pyogenes on cultures. There was no abscess or evidence of necrotising fasciitis intraoperatively. The patient improved with intravenous antibiotics and was stepped down from the ICU after four days and discharged on day eight. One month post admission there was significant superficial skin loss to both ventral and lateral aspect of the penis, with healthy granulation tissue at the base. The patient opted for conservative management with regular dressings. He reported normal sexual and urinary function three months post admission.

**Conclusion:**

This is the first published case of sepsis from a penile infection in the context of hyaluronic acid penile fillers. In an era of escalating demand for penile cosmetic procedures, there is an increasing need for early recognition and appropriate management of penile filler infections. We report an unusual case of a localised penile infection rapidly progressing to sepsis with multi-organ failure requiring intensive care support. The case demonstrates early surgical intervention with targeted antimicrobials can result in successful eradication of infection, with satisfactory cosmetic and functional outcomes for patients.

## Background

Penile enhancement with injectable agents is a growing trend and yet has received limited scientific attention, despite the potential of serious complications. We report a case of severe penile cellulitis in a 31-year-old man who underwent subcutaneous penile hyaluronic-acid filler insertion. The localised infection rapidly progressed to septic shock requiring intensive care support and subsequent operative drainage. This is the first reported case of fulminant septic shock with multi-organ failure contributed by hyaluronic-acid penile fillers. The unusual severity and rapid progression of penile cellulitis in this case was multifactorial, due to penile fillers, abnormal scar tissue from previous hypospadias repair and behavioural risk-factors. This case demonstrates early surgical intervention in conjunction with appropriate antimicrobials and supportive care can result in satisfactory cosmetic and functional outcomes.

## Case presentation

A 31-year-old male presented to the emergency department with a 2-day history of progressive pain and swelling of the penile shaft. This was preceded by unprotected sexual intercourse three days prior. The patient subsequently self-administered a single 50 mg dose of oral prednisone the following morning and subsequently developed subjective chills on the same day.

Relevant past medical history included subcutaneous hyaluronic filler injections at a cosmetic clinic for penile girth enhancement two months prior to presentation, distal tubularised hypospadias repair and genital herpes simplex. The patient had multiple behavioural risk factors, including regular self-administered intra-muscular testosterone injections, self-injection of growth hormone to the base of the penis (performed one month prior to penile filler procedure), multiple heterosexual sexual partners, cocaine use, heavy alcohol consumption (22 standard drinks/day) and a 10 pack-year smoking history.

**Fig. 1:**
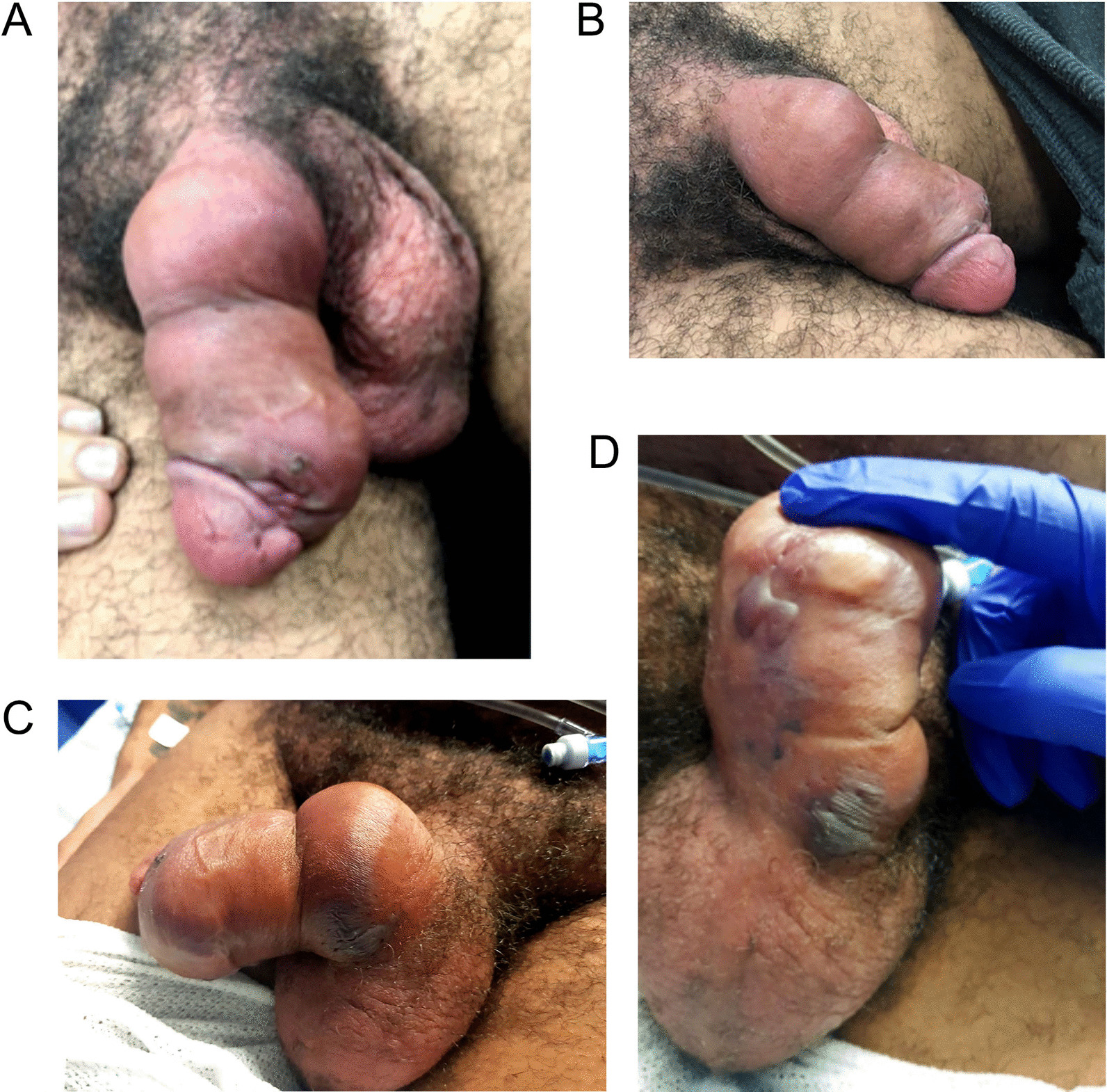
Penile swelling and erythema on presentation. **A**, **B** Gross penile oedema and erythema with an abrasion on distal ventral penile shaft. Images taken on presentation to Emergency Department. **C**, **D** New blistering overlying cosmetic filler injection sites. Image taken seven hours from initial presentation

On arrival, the patient was tachycardic to 127, hypotensive to 90/50 and febrile to 40 °C. Physical examination revealed gross penile oedema and erythema of the penile shaft ceasing at the base of the penis, with an associated curvature of the penile shaft towards the 8 o’clock position. There was also a small tender abrasion on the distal ventral aspect of the penile shaft 1 cm proximal to the glans (Fig. 1A). Blood tests showed a white cell count (WCC) of 6.1 × 10^9^/L, creatinine of 95 μmol/L and lactate of 2.3 mmol/L. Urinalysis was negative. The patient was admitted and commenced on intravenous Flucloxacillin for presumed penile cellulitis.

**Fig. 2:**
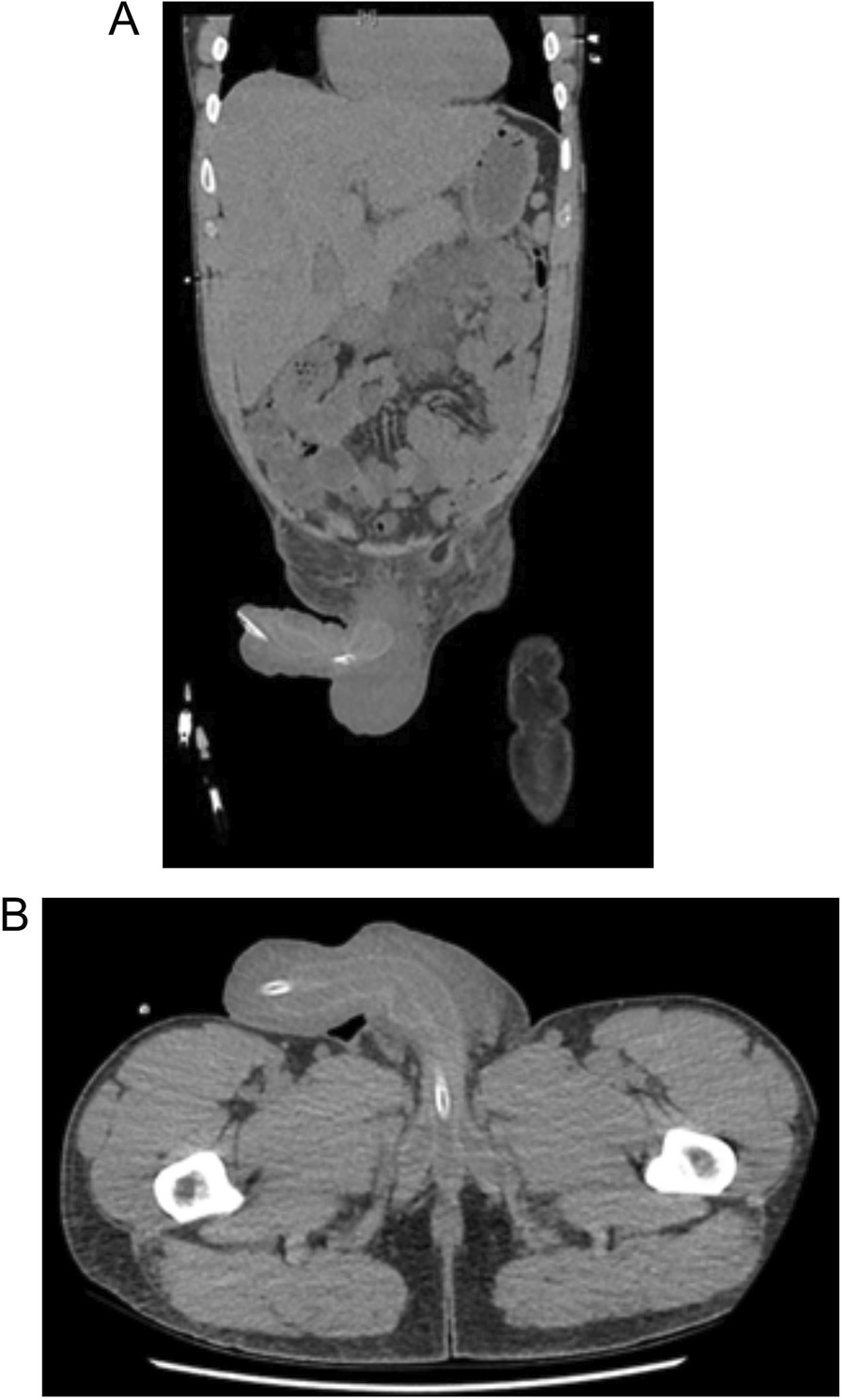
CT scan of the abdomen and pelvis. **A** Coronal and **B** Axial sections showing indwelling catheter in situ with extensive penile and pre-pubic soft tissue swelling. No identified abscess or gas locules

Overnight the patient became increasingly drowsy with a persistent fever > 40 °C and tachycardia. Repeat examination showed new blistering of the penile skin overlying the penile fillers (Fig. 1B). Repeat blood tests showed marked changes with WCC of 25 × 10^9^/L, creatinine of 225 μmol/L and lactate of 6.4 mmol/L, in less than seven hours from initial presentation. He was subsequently transferred to ICU for vasopressor and inotropic support. Antibiotics were changed to intravenous Meropenem, Clindamycin and Vancomycin as empiric therapy towards possible Fournier’s Gangrene, on advice from Infectious Diseases specialists. A subsequent Computerised Tomography (CT) of the abdomen and pelvis showed penile oedema and stranding, however excluded intra-pelvic abscess or gas locules (Fig. 2).

The patient was transferred to the operating theatres and an incision was made over the largest blistered site on the penile shaft, which revealed a pus stained filler above Buck’s fascia. This filler was removed, albeit with resistance requiring manual pressure to express the filler. The tissue adjacent to the infected filler was inflamed but no further debridement was required. A 21G needle was used to pierce the blistered skin overlying the penile fillers to facilitate drainage of the remaining penile fillers. The wound was dressed with an alginate dressing surrounded by absorbent gauze.

Penile swabs were positive for Streptococcus Agalactiae, Anginosus and Pyogenes, all of which were penicillin sensitive. Operative sample of the penile filler grew Streptococcus Pyogenes (penicillin sensitive). The patient was switched to intravenous Benzylpenicillin on day three on advice from Infectious Disease specialists, and dressings were changed daily. He was weaned off vasopressors and stepped down from ICU on day four.

The patient was subsequently counselled on safe sexual practices and received appropriate drug and alcohol counselling for his polysubstance abuse. A sexually transmitted infection screen showed active intercurrent Herpes Simplex Virus 2 infection for which he was commenced on oral Valaciclovir whilst in hospital. This was decreased to a prophylactic dose on discharge whilst his wounds healed. He was stepped down to oral Amoxicillin on day eight with a plan for three weeks of total antibiotic treatment. On discharge the blisters has flattened with no remaining fluid present, and the penile oedema and erythema had significantly improved.

**Fig. 3:**
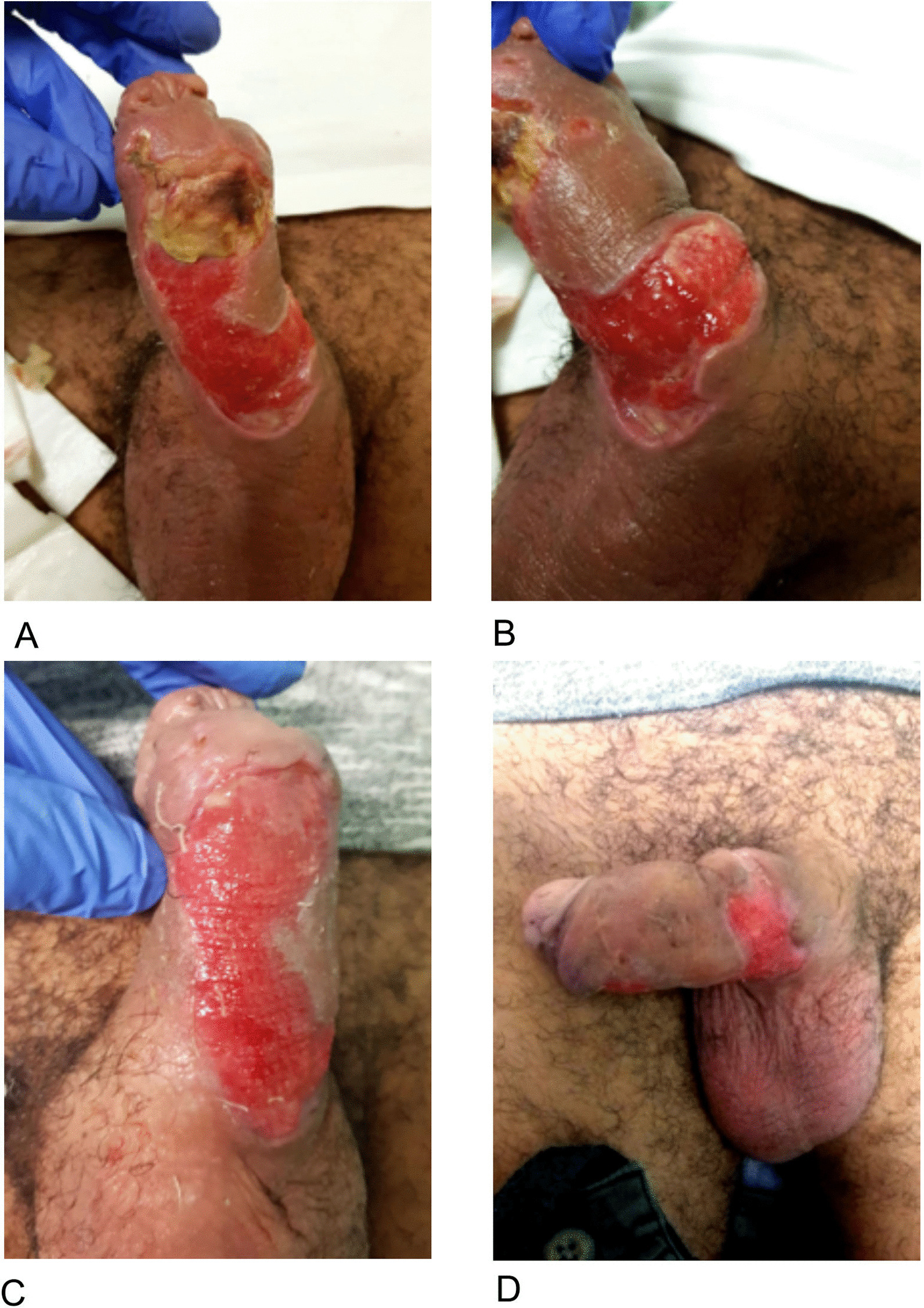
Follow-up of penile wounds. **A** 2 weeks post discharge, fibrinous tissue at distal edge with otherwise healthy tissue base. **B** 2 weeks post-discharge, induration at base of penis with scant purulent discharge expressed. **C**, **D** 5 weeks post discharge, significant improvement in inflammation with remaining superficial skin loss

At his follow up 2-weeks post-discharge, there was superficial skin loss to both the ventral and lateral aspect of the penis, corresponding to the initial blisters overlying his penile fillers with healthy granulation tissue at the base. A small one centimetre area of fibrinous tissue remained at the distal aspect of the shaft (Fig. 3A). There was also a small area of induration at the peno-scrotal junction (Fig. 3B), which expressed a scant amount of purulent discharge that was culture-negative. There was no palpable abscess and ultrasound excluded further penile or scrotal collection.

At his second follow up at five weeks post-discharge, the patient reported ongoing clinical improvement and normal erectile function. Examination revealed significant reduction in the inflammation at the base of penis with no remaining induration. The base of his wounds continued to appear healthy (Fig. 3C, D). The patient was reviewed by a Plastic Surgeon and was offered a split skin graft but was declined by the patient in preference for regular dressings. At three months post-discharged, the patient reported normal sexual and urinary function, and ongoing wound improvement.

## Discussion and conclusion

In an era of growing emphasis on physical appearance, there is an increasing demand for penile augmentation procedures [1, 2]. The goal of these procedures is a symmetrical increase in penile girth in both flaccid and erect states [3]. Most men who seek these procedures have physiologically normal penises and undertake these procedures for cosmetic and psychological reasons [1]. Currently there is no recommended indication nor proposed guidelines for penile girth enlargement [2]. The American Urological Association and Sexual Medicine Society of North America have both issued policy statements indicating there is insufficient data to substantiate the safety or efficacy of penile lengthening or girth enhancement surgery [4, 5].

Within the field of penile girth enhancement, there is an increasing utilisation of penile subcutaneous injection therapy as it is seen as simple, safe and minimally invasive [2, 3]. In this case hyaluronic acid, a long-lasting resorbable dermal filler was used. The filler is typically injected in small aliquots above Buck’s fascia with a 21G needle after application of topical local anaesthetic cream (lignocaine 25 mg/g and Prilocaine 25 mg/g) and an intra-cavernosal injection to induce a chemical semi-erection [2].

While there is evidence of hyaluronic acid use in cosmetic facial dermatology, evidence of its use in penile girth enhancement is limited. Currently there is no U.S. Food and Drug Administration (FDA) approved injectable filler for the penis, nor is there a review of complications from penile fillers in the literature. However a case series of soft-tissue fillers highlight important cosmetic, functional and systemic complications, which can lead to complex penile reconstructive surgery (Table 1) [1, 3, 6]. Fillers used in this study include hyaluronic acid, polymethyl-methacrylate injection, silicone and autologous fat [1, 3, 6].

**Table 1:** Complications of penile filler injections

Cosmetic	Penile distortion, abnormal curvature and asymmetry [3] Nodules—liquified, necrotic, calcified [6] Reabsorption, drifting or distant migration [7] Granulomatous reactions [8]
Functional	Peyronie’s disease [3] Erectile dysfunction [6] Male dyspareunia [6] Decreased sensation [9]
Systemic	Venous occlusion and acute hypersensitivity reaction [10] Fat embolism leading to cardiopulmonary collapse and death [11] Silicon embolism, pneumonitis, multi-organ failure and death [12, 13]

The role of injectable fillers as a potential immunological barrier and medium for bacterial growth has been established in cosmetic facial hyaluronic acid injections, with a 1% risk of an acute or delayed infection [14]. Dermal facial filler infections can present as acute inflammation or abscess at the site of injection and are typically due to common skin pathogens such as Staphylococcus Aureus or Streptococcus Pyogenes. Delayed or chronic infections tend to affect a more generalised area and may involve atypical organisms such as Escherichia Coli [15]. These infections are related to the formation of bacterial biofilm, which is an aggregation of bacterial cells embedded in a matrix of bacterial macromolecules on the surface of fillers [14].

Eradication of biofilm bacteria is notoriously difficult due to their growth pattern, emergence of resistant phenotypes and resistance to antibiotic penetration. Bacteria within the biofilm exist in a dormant state and can be activated by an immunosuppressed state, trauma, or iatrogenic manipulation [14, 15]. It is reasonable to conclude that penile hyaluronic acid fillers behaves similarly, with a greater likelihood of infection due to extensive bacterial flora and susceptibility to trauma in the urogenital region. This was demonstrated in our case with the appearance of pus-stained fillers intraoperatively and growth of Streptococcus Pyogenes, a common skin coloniser, on operative wound swabs and cultures. In this case, subcutaneous invasion of bacteria likely occurred during sexual intercourse through the abrasion (Fig. 1A) or a herpetic sore, which then invaded the hyaluronic acid fillers. It was considered less likely to be a delayed infection from the initial insertion of penile fillers, due to the onset of symptoms following unprotected intercourse.

Imaging has a limited role in the acutely deteriorating patient. In this case, CT was performed to exclude necrotising fasciitis or a deeper perineal or pelvic collection contributing to the patient's rapid deterioration to guide operative planning and consent. In dermatology, ultrasound is the imaging modality of choice for assessment of cosmetic fillers. High-density hyaluronic acid appears as well delineated, anechoic, pseudocystic structures without internal echoes. CT and MRI may provide limited information to gauge the extent of filler deposits, however do not specifically detect hyaluronic acid deposits [16].

Surgical intervention to remove the potentially infected penile fillers as ‘source control’ was advocated, given the patient’s rapid decline despite aggressive medical management. During pre-operative discussion with the patient’s initial cosmetic surgeon it was suggested that routine hyaluronic acid fillers are usually easily expressible after an incision, however, this was not evident due to the inflammatory response of the surrounding tissue. Due to the circumferential location of the penile fillers, there was concern regarding the long term cosmetic and sexual function if multiple incisions were performed. Hence the intra-operative approach was converted to a needle aspiration combined with manual pressure to remove the infected penile fillers.

The diagnostic challenge in these cases lies not in the identification of infection but the assessment of the likely severity and trajectory. Most cases of penile cellulitis rapidly respond to antibiotic therapy [17]. However, there is potential for rapid deterioration, as demonstrated in this case, where scarring from previous hypospadias repair, combined with hyaluronic acid penile fillers contributed to the severity of infection. Furthermore, the patient’s self-administration of prednisone and history of polysubstance abuse likely contributed to a compromised immune state. Indeed active polysubstance abuse in patients undergoing inflatable penile prosthesis surgery has been shown to significantly increase risk of postoperative infection [18]. Clinicians need to assess patient’s social history beyond medical comorbidities to adequately assess and predict infection severity.

In an era of growing emphasis on physical appearance and demand of injectable fillers, cosmetic filler infections is more than ever, important to recognise and manage appropriately. There is likely an underestimation of the complications from penile girth enlargement due to a lack of available data. This is the first published case of sepsis from a penile infection contributed by hyaluronic acid penile fillers. In this case, the localised infection rapidly progressed to sepsis with multi-organ failure requiring intensive care support and operative source control. The unusual severity of the case was due to a combination of host risk-factors and hyaluronic-acid penile fillers acting as a foundation for bacterial biofilm formation. This case demonstrates early surgical intervention with targeted antimicrobials can result in successful eradication of infection and satisfactory cosmetic and functional outcomes for patients.

## Data Availability

Data sharing is not applicable to this article as no datasets were generated or analysed during the current study.

## Abbreviations

WCC: White cell count
CT: Computerised tomography
FDA: Food and Drug Administration
